# The Effect of Blood Flow Restriction Training on Quadriceps Muscle Strength and Functional Performance Following Isolated Anterior Cruciate Ligament Reconstruction: A Pilot Study

**DOI:** 10.7759/cureus.79821

**Published:** 2025-02-28

**Authors:** Suzanna M Ohlsen, Mia S Hagen, Kathleen Cummer, Scott Telfer, Majid Chalian, Albert O Gee, Christopher Y Kweon, Kenneth M Chin, Cristine Agresta

**Affiliations:** 1 Orthopaedic Surgery, University of Washington Medical Center, Seattle, USA; 2 Physical Medicine and Rehabilitation, University of Washington Medical Center, Seattle, USA; 3 Radiology, University of Washington Medical Center, Seattle, USA

**Keywords:** acl rehabilitation, anterior cruciate ligament (acl) reconstruction, blood flow restriction therapy, blood flow restriction training, musculoskeletal mri

## Abstract

Background: Quadriceps weakness is common after anterior cruciate ligament (ACL) injuries and ACL reconstruction (ACLR). Blood flow restriction (BFR) training is being increasingly used during ACLR rehabilitation protocols to facilitate a hypoxic cellular environment that triggers a local stress response theorized to promote muscle hypertrophy, and thus muscle strength, without mechanically loading a healing ACL graft. While BFR is a popular addition to therapy, scientific methods used to examine BFR training following ACLR have been inconsistent or insufficient to determine whether early BFR has a significant therapeutic effect on strength and function, and few studies have used advanced imaging to assess changes in muscle volume and composition.

Purpose: This study aimed to investigate changes in postoperative quadriceps strength and muscle volume between conventional ACLR rehabilitation with early BFR training and conventional ACLR rehabilitation with sham (e.g., non-therapeutic pressure) BFR training. We secondarily sought to evaluate the effect of early BFR training on late-stage functional and patient-reported outcomes (PROMs).

Methods: Ten individuals with a unilateral isolated ACLR were randomized to receive 200 minutes of BFR or sham (CON) training as part of their ACLR rehabilitation protocol. Quadriceps and hamstring strength were taken via a handheld dynamometer to calculate limb symmetry indices (LSI) at eight and 36 weeks postoperatively. Magnetic resonance (MR) images were acquired of the bilateral knees pre- and post-BFR or CON training and evaluated for muscle volume and adipose composition. Single-leg hop tests were performed at the conclusion of the rehabilitation protocol around 36 weeks postoperatively. PROM measures were measured by the International Knee Documentation Committee (IKDC) and Knee Injury and Osteoarthritis Outcome Score (KOOS) at baseline and eight and 36 weeks postoperatively.

Results: At eight weeks postoperatively, there was not a significant difference in quadriceps and hamstring LSI between the BFR and CON groups. At 36 weeks postoperatively, there was a significant increase in strength within all groups, but there were no significant differences in the improvement of either quadriceps or hamstring strength between the BFR and CON groups. There was no significant change in quadriceps intramuscular adipose composition or muscle volume between pre- and post-BFR MR images within the BFR group. There was additionally no difference between PROMs and adverse events between the two groups at the eight- and 36-week postoperative time points, and there were no complications with early BFR use.

Conclusion: In this pilot study, quadriceps and hamstring strength, muscle volume, and intramuscular adipose were not impacted over time or between the BFR and standard-of-care groups. Early BFR utilization had no effect on PROMs between BFR and standard of care as measured by IKDC and KOOS. Larger studies are needed to better understand the potential effects of early BFR on patient rehabilitation after ACLR.

## Introduction

Anterior cruciate ligament (ACL) injuries and ACL reconstruction (ACLR) are associated with protracted quadriceps weakness and disuse atrophy [1,2]. It is common to have prolonged quadriceps weakness following ACLR despite extensive rehabilitation, and, for some, these deficits can persist for years and affect long-term function [1-3]. Because poor and persistent quadriceps function results in short- and long-term negative consequences, much of ACLR rehabilitation is centered on resolving this strength impairment. Treatments like electrostimulation and electromyographic feedback, strength training, and neuromuscular re-education have been used to increase quadriceps strength following ACLR [3-5]. Of these, eccentric quadriceps strength training had the greatest improvement in strength. However, despite improvements, complete recovery and full resolution of quadriceps weakness were not achieved within the first year after surgery [6]. The lack of full recovery with eccentric strength training suggests that mechanical load achievable with traditional training methods following ACLR may be an insufficient stimulus for the desired adaptation in the quadriceps muscle.

Blood flow restriction (BFR) is increasingly being used as an intervention to build strength and muscle mass by mimicking heavy load resistance training by applying a tourniquet around the thigh to restrict arterial inflow while occluding venous return during exercise [7]. The resultant transient hypoxia mediates the release of metabolites and cytokines that promote vasodilation, angiogenesis, protein anabolism, and production of hematopoietic progenitor cells. This ultimately has been shown by some studies to facilitate muscle hypertrophy and increased strength without the inherent risks that would be conferred by the utilization of high-load resistance training during early ACL rehabilitation before the graft has healed [4,7-11]. Despite these reported benefits, there have been inconsistent outcomes reported in the literature and no clear consensus on the role of BFR in ACLR rehabilitation [4,12,13]. It is unclear if adding BFR training improves quadriceps strength and volume, function, and patient-reported outcome measures (PROMs) compared to traditional protocols.

The primary aim of this pilot study was to compare changes in postoperative quadriceps strength between conventional ACLR rehabilitation with early BFR training and conventional ACLR rehabilitation with sham (e.g., non-therapeutic pressure) BFR training, hereby referred to as the BFR and CON (control) groups, respectively. We hypothesized that those who received early BFR training in addition to ACLR rehabilitation would demonstrate a greater (positive) change in quadriceps strength limb symmetry and larger muscle volume at eight weeks postoperatively compared to those who did not receive BFR training. A secondary aim was to determine the effect of adding early BFR training on late-stage (36-week) functional outcomes using standard performance tests and PROMs. We hypothesized that those who received early BFR training would demonstrate better scores on functional hop tests and PROMs.

## Materials and methods

This study was conducted at the University of Washington Medical Center after obtaining approval from the University of Washington IRB Committee B (approval number: FWA #00006878).

Participant identification 

**Figure 1:**
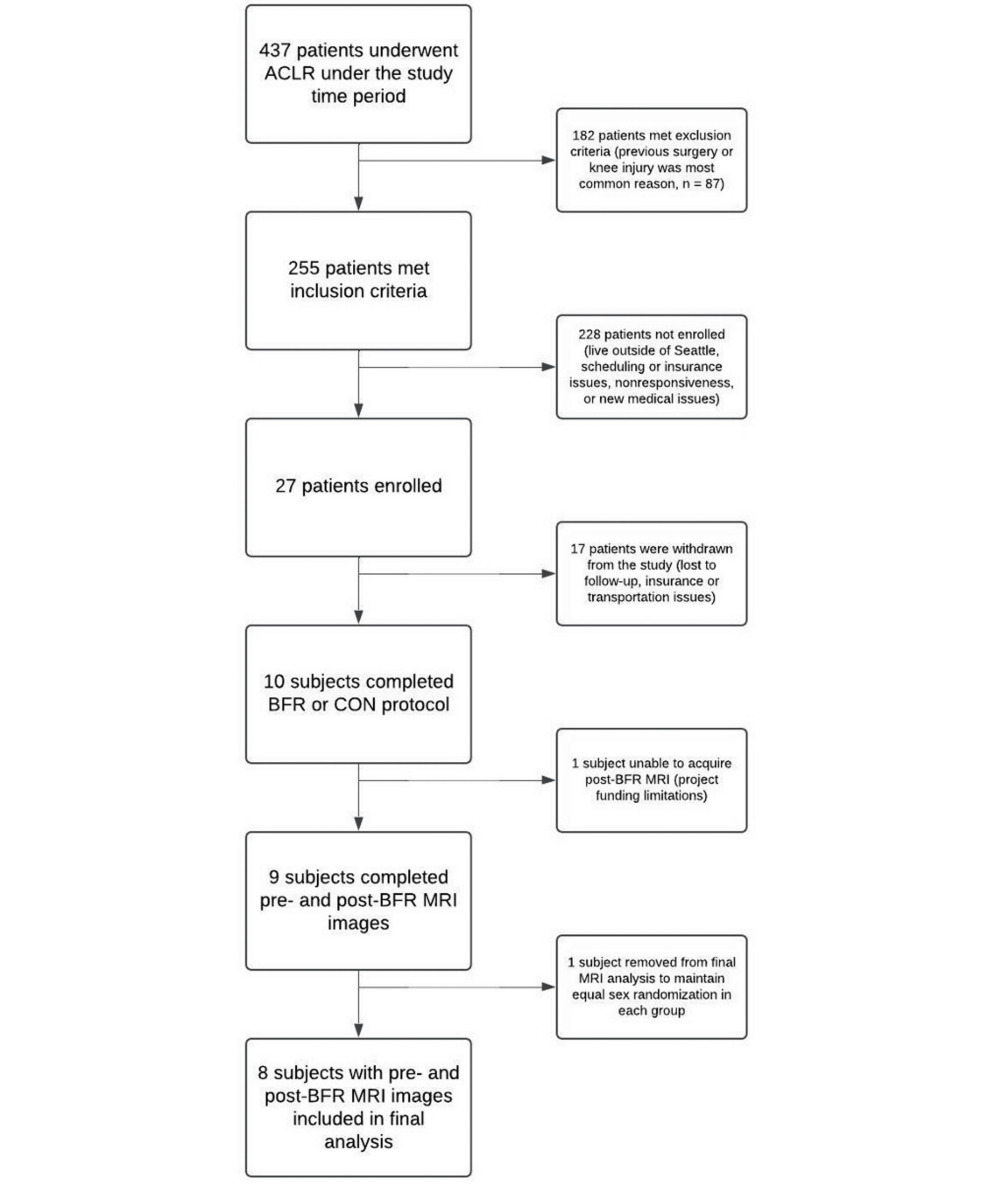
CONSORT flow diagram showing the selection of cohorts. Four hundred and thirty-seven patients underwent ACLR over the study time period, with 255 patients meeting the inclusion criteria for the study and 27 subjects enrolled. Seventeen of the enrolled subjects were withdrawn. A total of 10 subjects completed the BFR and CON ACLR rehabilitation protocols. Of these, nine patients acquired post-BFR images CONSORT: Consolidated Standards of Reporting Trials; ACLR: anterior cruciate ligament reconstruction; BFR: blood flow restriction; CON: control; MRI: magnetic resonance images

Potential participants were identified during their preoperative visit to our institution from November 1, 2020, to December 31, 2022. Administrative staff provided information about the study during patient care visits. Patients interested in study participation contacted the study team independently for possible enrollment. Interested patients were enrolled in the study if they met the following inclusion criteria: (1) age between 14 and 50 years old, (2) planned isolated unilateral ACLR procedure without meniscus repair, where the injury occurred within six weeks of the planned surgery date, and (3) plan to attend physical therapy at one of two participating clinics within our institution with physical therapists certified in the use of BFR. We excluded patients with any of the following: (1) history of severe lower extremity injury or previous knee surgery of either leg, (2) prior use of BFR training, (3) presence of severe or uncontrolled hypertension, (4) current or prior history of cardiovascular or pulmonary conditions, or (5) high risk for deep vein thrombosis as defined by a score of ≥3 on the Wells Prediction Rules. A Consolidated Standards of Reporting Trials (CONSORT) flow diagram depicting the selection of cohorts is shown in Figure 1. 

Approval was obtained prior to data collection. All participants provided their written informed consent prior to participation. Participants' baseline demographics of age at the date of surgery, sex, and body mass index (BMI) were collected and are presented in Table 1. 

**Table 1:** Demographics and characteristics of training between the BFR and CON (sham BFR) groups. Values are in mean (SD) with min-max range. The BFR training (therapeutic and sham) was accompanied by standardized physical therapy exercises and a daily home exercise protocol BFR: blood flow restriction; CON: control; BMI: body mass index; HT: hamstring tendon; QT: quadriceps tendon; BPTB: bone-patellar tendon-bone; Allo: allograft; AOP: arterial occlusion pressure; RPE: rate of perceived exertion during physical therapy, measured on a scale of 6-20

Demographics	BFR	CON	P-value
Age (years)	26.6 (9.8)	30.4 (10.1)	0.28
BMI (kg/m^2^)	22.7 (3.5)	28.6 (5.3)	0.04
Graft type	3 HT, 1 QT, 1 BPTB	1 HT, 3 BPTB, 1 Allo	-
Total sessions (no)	12.2 (2.8)	12.1 (2.0)	0.39
Days since surgery at the start of BFR/CON training	16.6 (3.8)	18.9 (6.2)	0.33
Days since surgery at the end of BFR/CON training	70.2 (21.6)	82.6 (26.6)	0.46
Inflated cuff time (min)	16.5 (5.1)	17.3 (5.4)	0.21
Max AOP (mmHg)	181.1 (23.3)	179.0 (34.9)	0.58
Treating AOP (mmHg)	142.7 (19.1)	35.6 (7.0)	<0.01
Treating AOP (%)	78.9 (4.1)	20.1 (0.5)	<0.01
RPE (6-20)	13.9 (2.9)	12.9 (2.5)	0.01

Experimental protocol 

Participants were block-randomized by sex to either the sham (CON) or the BFR training (BFR) group. Both groups received a standardized protocol of exercises and inflated cuff time criteria (200 minutes in total throughout the duration of rehabilitation) that only differed by cuff occlusion pressure. Occlusion pressure was controlled per group and per patient using a personalized (versus standard tourniquet) pressure cuff (Delfi Personalized Tourniquet System, Owens Recovery Science, San Antonio, Texas, United States). The CON group had an inflated cuff occlusion pressure of approximately 20% arterial occlusion pressure (AOP) representing sham cuff training, whereas the BFR group had an inflated cuff occlusion pressure of between 60% and 80% AOP (Table 1). Participants received BFR or CON training as part of their regularly scheduled physical therapy sessions which were provided to both groups by one of the 14 licensed physical therapists who was certified in BFR. Physical therapy with BFR or CON training began two weeks after surgery, and visits were in-person two times per week for six weeks, with an at-home exercise program. BFR therapy protocols are outlined in Table 2. The BFR and CON training ended after subjects completed 200 minutes of exercise using the pressurized cuff. Patients were asked their rate of perceived exertion (RPE) on the Borg scale during their therapy session. Physical therapists were not blinded to the subject group as the therapist had to set the appropriate occlusion pressure for each group. 

**Table 2:** BFR protocol given to the physical therapists treating the study participants BFR: blood flow restriction; CON: control; AOP: arterial occlusion pressure; ACLR: anterior cruciate ligament reconstruction

BFR protocol					
Begin protocol when…					
(a) The patient can complete 1-3 straight leg raises without a quadriceps lag sign (BFR cannot be used prior to this phase)					
(b) Knee range of motion is 0-70 degrees					
(c) The patient is 7-21 days from surgery. Patients who are >4 weeks postoperative from ACLR are ineligible					
Follow the criterion-based progression stated below:					
Phase 1 (weeks 1-2; postop weeks 3-4)	Progress when…	Phase 2 (weeks 3-4; postop weeks 5-6)		Progress when…	Phase 3 (weeks 5-6; postop weeks 7-8)
(1) Terminal knee extension (TKE)	Able to complete full repetitions (30-15-15-15)	(1) Straight leg raise (SLR) with load		Able to complete full repetitions (30-15-15-15)	(1) Unilateral leg press
					(2) Single-leg squat
					(3) Step up (box height approx. 7 in., minimum knee flex 45° and progress towards 90°)
(2) Isometric quad set		(2) Long-arc quad (90-40° or as per surgical protocol)			
(3) Straight leg raise (SLR) unloaded					
		(3) Unilateral leg press			
BFR-specific requirements:					
(a) AOP: BFR=60-80%; CON=20%					
(b) Session AOP may be changed during the session according to patient tolerance					
(c) BFR group AOP should not drop below 60% or be raised above 80%					
(d) Reps: 30-15-15-15, complete as many of these repetitions as tolerated by the patient					
(e) Rest periods: 30-60-second rest between sets; 2-minute rest between exercises					
(f) Frequency: 2 times per week					
(g) Duration: occurring between two and eight weeks postop from ACLR					
(h) Sessions: 12 (total)					
(i) Session cuff time should be approximately 20-30 minutes					
(j) BFR can be performed at any time during the treatment session but we ask that it is blocked such that all exercises listed occur consecutively					
Terminate protocol when…					
(a) The patient has reached a minimum of 200 minutes of total inflated cuff time					
Protocol guideline dos and don'ts:					
Dos			Don'ts		
Do answer patient's questions to the best of your ability without unblinding the patient. If unable to answer questions, please refer the patient to a study team member			Do not tell the patient which treatment group they are in. If the patient knows which group they are in, it will unblind the patient, leading to bias within the study. We will not be able to objectively evaluate their data		
			Do not continue with BFR after the protocol is completed and do not use BFR with any patient in the control group. This will negate the purpose of the study		

Assessment of strength

**Figure 2:**
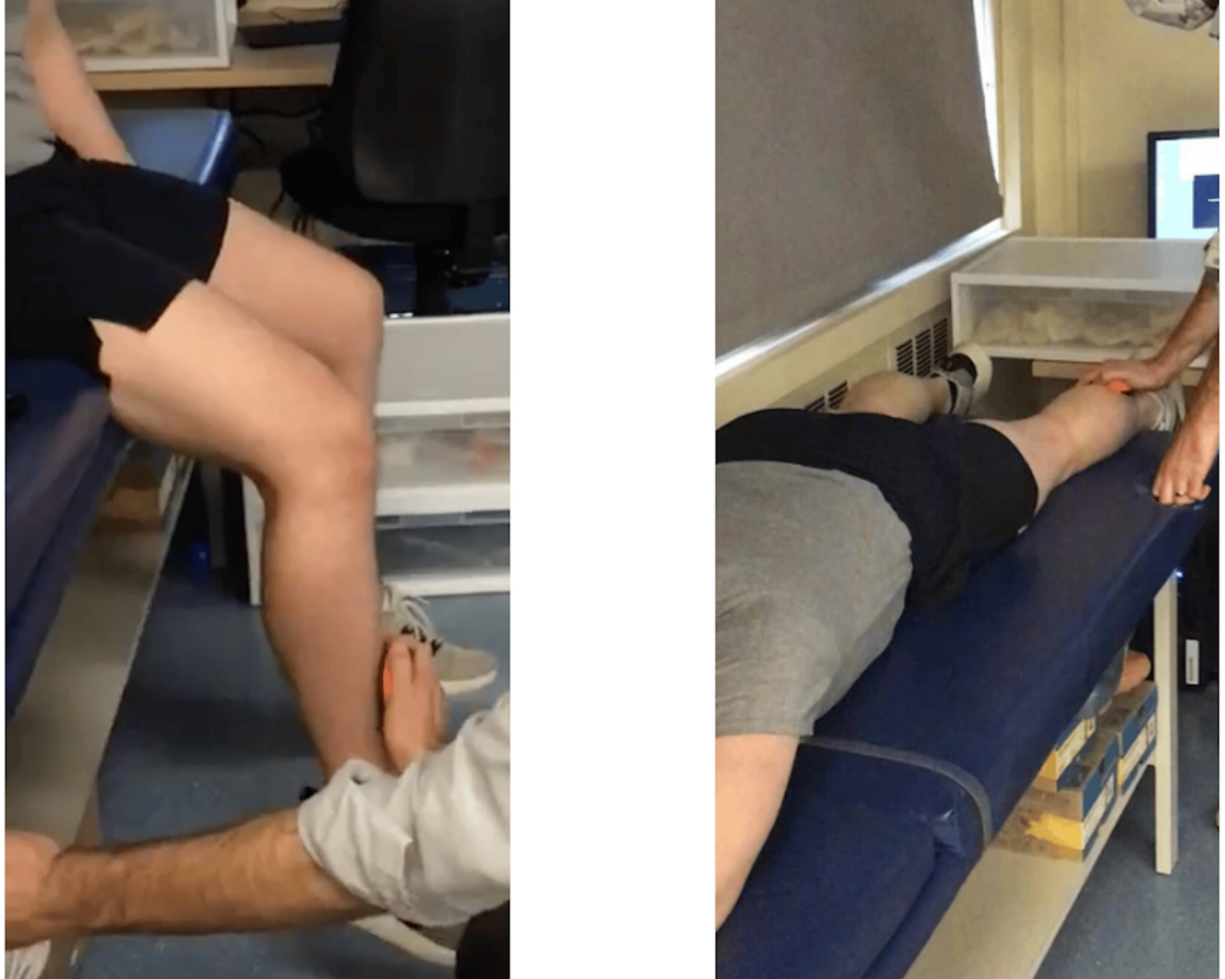
(Left) Maximum voluntary isometric strength of the knee extensors using a handheld dynamometer. (Right) Maximum voluntary isometric strength of the knee flexors using a handheld dynamometer

Strength measures were collected using a handheld dynamometer (Activ5, Anybody, Inc., San Diego, California, United States), a valid, low-cost, and user-friendly method for monitoring strength after ACLR [14,15]. Dynamometer testing was performed on all subjects after BFR training had concluded (approximately eight weeks postoperatively) and around 36 weeks postoperatively. Protocols are shown in Table 2. Maximum voluntary isometric strength of the knee extensors and flexors were collected. For isometric knee extension, subjects were seated on a chair with their hip and knee joints each at 90° flexion. The handheld dynamometer was first placed at the distal portion of the anterior tibia of the unaffected limb (Figure 2). The subject was instructed to extend their leg against the device at maximum force for five seconds. This was repeated three times with a 30-second break in between each trial. Once three trials were completed, the patient was then tested on the ACLR limb. For isometric knee flexion, subjects laid prone with their hip and knee joints each at 0° flexion. The handheld dynamometer was first placed at the distal portion of the posterior tibia of the unaffected limb (Figure 2). The subject was instructed to flex their leg against the device at maximum force for five seconds. This was repeated three times with a 30-second break in between each trial. Once three trials were completed, the patient was then tested on the ACLR limb. Limb symmetry indices (LSI) were calculated with the following formula, using the mean value from the three completed trials: LSI=(ACLR limb/unaffected limb)×100.

Functional performance

Functional performance was measured at 36 weeks postoperatively for all subjects who completed the full 200 minutes of BFR training. Subjects performed a series of single-leg hops commonly used in return-to-sport testing, including (1) single-leg hop for distance, (2) triple hop, and (3) six-meter timed hop [16]. These were measured on both the ACLR and contralateral limbs to control for a subject's baseline value. LSI for hop testing were calculated between the ACLR and contralateral limbs, using the formula listed above, for both the BFR and CON groups. 

PROMs

PROMs were analyzed for all 10 subjects at baseline and both eight and 36 weeks postoperatively. These included the International Knee Documentation Committee (IKDC) and Knee Injury and Osteoarthritis Outcome Scores (KOOS) for pain, function, sports and recreation, and quality of life measures. The IKDC is a validated, knee-specific measure of patient-reported outcomes of symptoms, function, and sports activity, with the goal of standardizing outcome measures and the benefit of being able to compare outcomes across a variety of knee pathologies [17]. Similarly, the KOOS is a validated tool developed as an extension of the Western Ontario and McMaster Universities Osteoarthritis Index (WOMAC) to measure patient-reported outcomes over five categories [18].

Magnetic resonance imaging (MRI) analysis 

**Figure 3:**
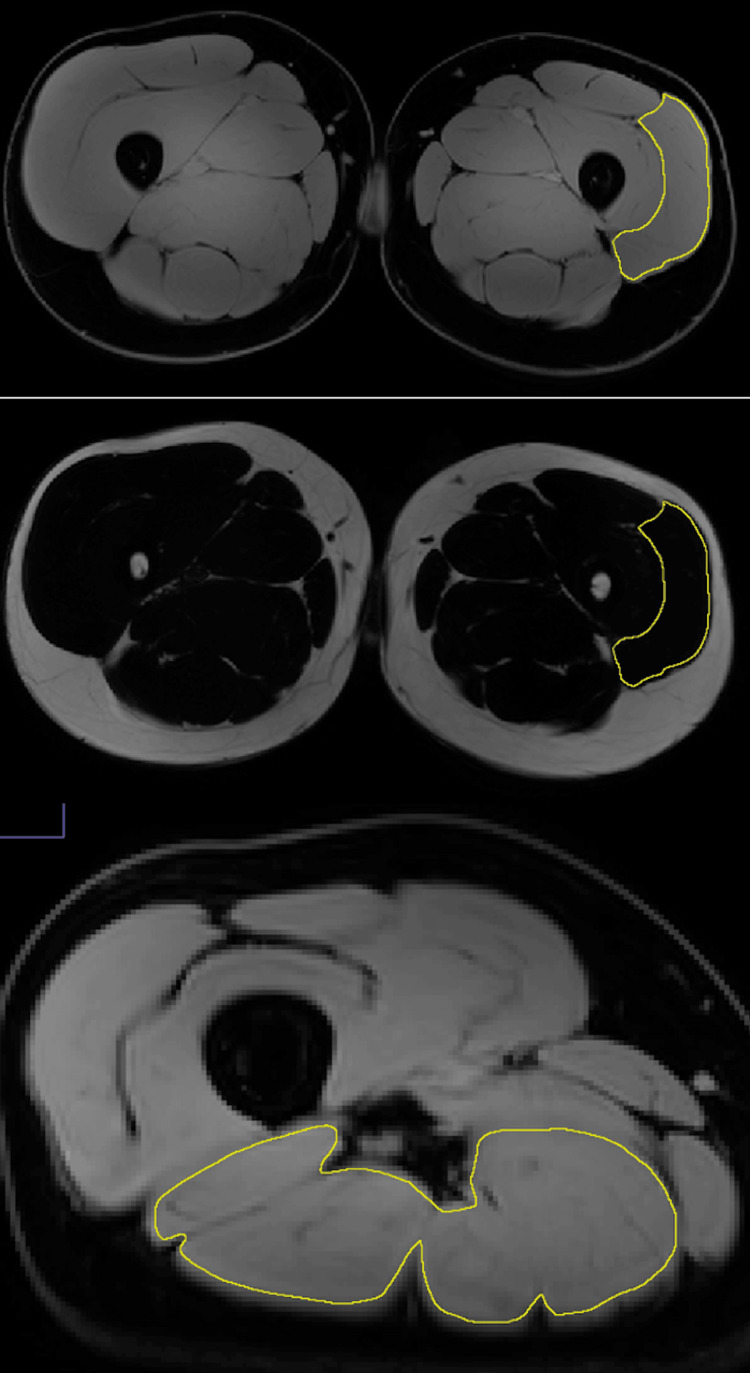
Magnetic resonance images with representative cuts demonstrating (A) ROI drawn on water phase image, (B) ROI copied to fat phase image, and (C) ROI excluding visible fascia and fat ROI: region of interest

All MRI scans were performed on an Ingenia Elition 3.0T X (Philips Healthcare, Cambridge, Massachusetts, United States) machine. MRI of the knee was acquired at two and eight weeks postoperatively. These time points were selected to best compare MRI of the knee pre- and post-BFR training. As a control, the contralateral knees of each subject were also scanned at both time points. Each study was acquired using axial Turbo Spin Echo (TSE) 3D Proton Density mDixon sequence with a two-point mDixon technique. Voxel size was 1×1 mm with layer thickness set to 8 mm and a slice gap of 0 mm. The matrix size was 450×300, with an echo time (TE) of 30 ms, a repetition time (TR) of 3000-5000 ms, and a flip angle of 90°. Images were analyzed by a senior radiology resident (E.H.) under the guidance of an attending musculoskeletal radiologist (M.C.), using ImageJ software, with anonymous patient identifiers and without any further clinical data. This method has been previously shown to have high intra-observer reproducibility [19]. Manual two-dimensional regions of interest (ROIs) were drawn on every third muscle slice for the following muscle groups, rectus femoris, vastus lateralis, vastus intermedius, vastus medialis, and hamstrings, as shown in Figure 3. The sum of the ROI cross-sectional area for each muscle was multiplied by 24 mm (to account for three slices×8 mm slice thickness) to calculate the approximate muscle volume. The entire muscle was analyzed for each subject, except for one subject who was taller than the scan range. For those studies, the inferior portion of the hamstrings and vastus medialis were not fully included. The intramuscular adipose tissue (IMAT) was calculated from the same ROIs using the following formula from Ogawa et al. [19]: IMAT=(100×fat ROI)/(fat ROI+water ROI).

ROIs were drawn on water phase images, as these had better muscle definition, and copied to fat phase images. Substantial visible intramuscular fascia and fat were excluded. A few areas of high deviation in the IMAT value were analyzed for single slice focal fat saturation artifact and excluded from the IMAT calculations as applicable. These image ROIs were not excluded from the muscle volume calculations. 

Statistical analysis 

Mean and standard deviations were calculated for outcome variables of interest. Paired t-tests were used to identify significant differences in demographics or training characteristics (in terms of total number of sessions, cuff time, time between protocol and surgery date, AOP, and RPE) between groups. The primary outcome variables of interest were LSI for (1) mean quadriceps strength, (2) mean hamstring strength, and (3) mean hamstring-to-quadriceps strength ratio. Variables were calculated for both time points, post-BFR training (eight weeks postoperatively) and 36 weeks postoperatively following ACLR. The changes in strength measures were also calculated between each time point, using ANOVA. Pre-BFR and post-BFR IMAT calculations from MRI were analyzed using ANOVA to compare differences in time, ACLR, and contralateral limb strength, as well as strength by muscle group. All continuous variables were expressed as the mean±SD; a two-tailed p<0.05 was considered to indicate statistical significance. We calculated sample size utilizing effect size from existing literature on the effect of BFR on quadriceps strength and found that we needed 12 participants in each group in order to be adequately powered [20]. All statistical analyses were performed using Stata Statistical Software: Release 16.1 (February 2020; StataCorp LLC, College Station, Texas, United States). 

## Results

During the enrollment period, we identified 437 patients who underwent ACLR, of which 255 of these met the inclusion criteria. Twenty-seven subjects were enrolled in our study, with 17 subjects who were withdrawn from the study at various time points because of unresponsiveness, subsequent insurance restrictions, difficulty with transportation to the designated therapy sites, or loss to follow-up (Figure 1). Ten subjects completed the full rehabilitation protocol including a total of 200 minutes of BFR training, with eight subjects completing both pre-BFR and post-BFR MRI acquisition. Characteristics of participants in the BFR and CON groups are represented in Table 1. Of the 10 subjects included, four were female (40%). Groups were similar in terms of mean age, but BMI was slightly higher in the CON group. ACLR graft types included four patellar tendon autografts, four hamstring autografts, one Achilles tendon allograft, and one quadriceps tendon autograft. Within the BFR group, there were three hamstring autografts, one quadriceps autograft, and one patellar tendon autograft, and within the CON group, there were one hamstring autograft, three patellar tendon autografts, and one allograft. 

The influence of BFR on the primary outcome of knee flexion and extension strength is presented in Table 3, with quadriceps and hamstring LSI measured on a dynamometer and reported in kilograms. While there were significant increases in strength over time for both hamstring and quadriceps muscle groups in both the BFR and CON cohorts between the eight- and 36-week postoperative time points, there were no significant differences in the improvement of quadriceps or hamstring strength between the BFR and CON groups. There was also no difference in hamstring-to-quadriceps ratio at either eight or 36 weeks postoperative between the BFR and CON cohorts.

**Table 3:** Strength in kilograms as measured by dynamometer readings for knee flexion and hamstring-to-quadriceps ratios. Measurements were taken at eight weeks and 36 weeks postoperatively, in both operative and nonoperative limbs to calculate the LSI LSI: limb symmetry index; BFR: blood flow restriction; CON: control

LSI by muscle group	8 weeks	36 weeks	Difference	P-value
Quadriceps LSI				
BFR	30.9 (9.6)	42.5 (7.5)	11.6 (15.3)	0.03
CON	34.8 (5.7)	48.7 (4.9)	13.9 (7.6)	<0.01
P-value	0.23	0.08	0.46	-
Hamstring LSI				
BFR	36.8 (3.9)	45.4 (3.6)	18.6 (6.3)	<0.01
CON	40.7 (7.0)	48.7 (5.5)	8.0 (9.5)	0.04
P-value	0.16	0.15	0.55	-
Hamstring-to-quadriceps ratio LSI				
BFR	1.3 (0.3)	1.1 (0.2)	-0.2	0.84
CON	1.2 (0.3)	1.1 (0.2)	-0.1	0.69
P-value	0.65	0.56	0.43	-

On MRI evaluation, there were no statistically significant differences between intramuscular adipose tissue of the hamstring muscle group between the BFR and CON groups in either the affected or unaffected limbs (Table 4). Of note, there was no difference noted for rectus femoris fat composition between pre- and post-BFR MR images within the BFR group (p=0.27), though the control group had decreased fat composition in the operative extremity (p=0.05). In comparing muscle volume, there were no significant differences between BFR and CON for any muscle group at any time point in either the affected or unaffected limb, as shown in Table 5. 

**Table 4:** Cross-sectional MRI analysis of intramuscular adipose tissue in regions of interest (expressed as a percentage), comparing the hamstrings, rectus femoris, vastus intermedius, vastus lateralis, and vastus medialis in the surgical and nonoperative limb of subjects, at both pre- and post-BFR (therapeutic and sham) time points (MRI approximately six weeks apart). Lower percentages represent less adipose tissue in the muscle. Statistical analysis revealed no differences between the BFR and CON groups at any time point (not shown) BFR: blood flow restriction; CON: control; MRI: magnetic resonance imaging

Muscle group	Operative limb				Nonoperative limb				
	Pre	Post	Change	P-value	Pre		Post	Change	P-value
Hamstrings									
BFR (n=4)	10.8 (0.7)	10.8 (1.6)	-0.01 (1.4)	0.50	8.5 (2.9)		6.6 (1.0)	-1.9 (2.2)	0.13
CON (n=4)	10.1 (1.8)	9.1 (2.0)	-1.0 (1.8)	0.24	8.5 (2.9)		8.0 (2.9)	-0.5 (2.7)	0.41
Rectus femoris									
BFR	10.2 (2.9)	9.3 (0.8)	-1.0 (2.1)	0.27	7.2 (1.2)	5.8 (1.2)		-1.4 (1.4)	0.08
CON	9.0 (1.2)	7.2 (1.4)	-1.8 (1.5)	0.05	8.6 (0.8)	6.5 (2.7)		-2.1 (2.1)	0.08
Vastus intermedius									
BFR	9.1 (1.6)	9.1 (0.7)	0.01 (1.1)	0.50	7.2 (1.6)		6.0 (1.3)	-1.2 (1.5)	0.15
CON	8.9 (1.2)	7.9 (1.8)	-1.0 (1.5)	0.19	8.7 (1.6)		6.0 (2.5)	-2.8 (2.4)	0.05
Vastus lateralis									
BFR	8.8 (1.4)	9.0 (1.0)	0.2 (1.2)	0.60	8.2 (1.7)		7.2 (1.0)	-1.0 (1.4)	0.18
CON	9.6 (0.3)	8.2 (1.4)	-1.5 (1.2)	0.04	9.2 (1.0)		8.1 (2.0)	-1.1 (1.6)	0.18
Vastus medialis									
BFR	9.6 (2.1)	8.1 (1.4)	-1.5 (1.8)	0.13	6.1 (2.2)		4.4 (1.5)	-1.7 (2.0)	0.12
CON	7.9 (2.0)	6.7 (2.4)	-1.2 (2.1)	0.23	5.9 (2.0)		5.5 (2.4)	-0.5 (2.0)	0.38

**Table 5:** Cross-sectional MRI analysis of muscular volume in ROIs (expressed in cm3), comparing the hamstrings, rectus femoris, vastus intermedius, vastus lateralis, and vastus medialis in the surgical and nonoperative limb of subjects, at both pre- and post-BFR/CON time points (MRI approximately six weeks apart) BFR: blood flow restriction; CON: control; ROI: region of interest; MRI: magnetic resonance imaging

Volume by ROI	Operative limb				Nonoperative limb			
	Pre	Post	Change	P-value	Pre	Post	Change	P-value
Hamstrings								
BFR	477.6 (178.2)	479.8 (210.2)	2.2 (180.4)	0.50	535.9 (195.0)	503.0 (130.5)	-33.0 (154.6)	0.61
CON	520.8 (133.6)	562.0 (127.9)	41.1 (123.1)	0.34	578.5 (116.3)	587.3 (136.3)	8.8 (117.4)	0.46
Rectus femoris								
BFR	157.3 (51.4)	146.2 (55.9)	-11.2 (50.1)	0.61	164.3 (49.2)	160.1 (43.2)	-4.2 (42.9)	0.55
CON	163.1 (43.8)	166.0 (56.3)	3.0 (46.8)	0.47	194.5 (37.9)	206.2 (38.7)	11.7 (36.0)	0.34
Vastus intermedius								
BFR	251.9 (80.8)	244.6 (112.8)	-7.5 (90.9)	0.54	291.0 (72.2)	304.5 (93.1)	13.5 (77.5)	0.41
CON	280.6 (119.3)	285.5 (143.8)	5.0 (122.4)	0.48	337.0 (116.6)	380.6 (119.5)	43.6 (111.8)	0.31
Vastus lateralis								
BFR	354.4 (95.3)	336.5 (129.9)	-18.0 (105.9)	0.58	448.4 (122.3)	464.5 (162.9)	16.1 (133.6)	0.44
CON	359.4 (137.9)	386.1 (175.0)	26.8 (146.6)	0.41	466.7 (150.0)	485.7 (157.8)	19.0 (142.9)	0.43
Vastus medialis								
BFR	242.2 (65.0)	230.9 (83.6)	-11.3 (69.6)	0.58	285.6 (85.8)	313.3 (125.2)	27.7 (100.4)	0.36
CON	272.8 (126.0)	297.6 (163.8)	24.8 (135.9)	0.41	370.3 (131.6)	409.0 (129.5)	38.7 (122.6)	0.35

Incomplete data existed for functional testing as measured by time to perform six-meter hop and distance traveled for single-leg hop and single-leg triple hops, as some subjects were not able to complete all single-leg hop tests at the 36-week postoperative time point. Of the subjects who completed single-leg hop tests (n=8 for six-meter hop, n=7 for single-leg hop, and n=6 for single-leg triple hops), there were no observed differences between the BFR and CON groups as calculated by LSI (Table 6).

**Table 6:** Functional performance metrics analysis of patients who completed strength testing at eight and 36 weeks postoperatively: Six-meter hop is measured in seconds required to for a subject to complete single-leg hops for a distance of six meters. Hop for distance (distance a subject was able to hop on a single leg) and triple hop (distance a subject was able to travel with three sequential single-leg hops) are measured in centimeters. Incomplete data is available as some subjects were unable to complete all single-leg hop tests LSI: limb symmetry index; BFR: blood flow restriction; CON: control

Functional test	LSI
Six-meter hop (s)	
BFR (n=5)	119.1 (31.5)
CON (n=3)	135.6 (11.5)
P-value	0.79
Hop for distance (cm)	
BFR (n=3)	96.3 (2.8)
CON (n=4)	91.8 (11.9)
P-value	0.28
Triple hop for distance (cm)	
BFR (n=3)	104.3 (12.9)
CON (n=3)	83.8 (14.1)
P-value	0.07

**Figure 4:**
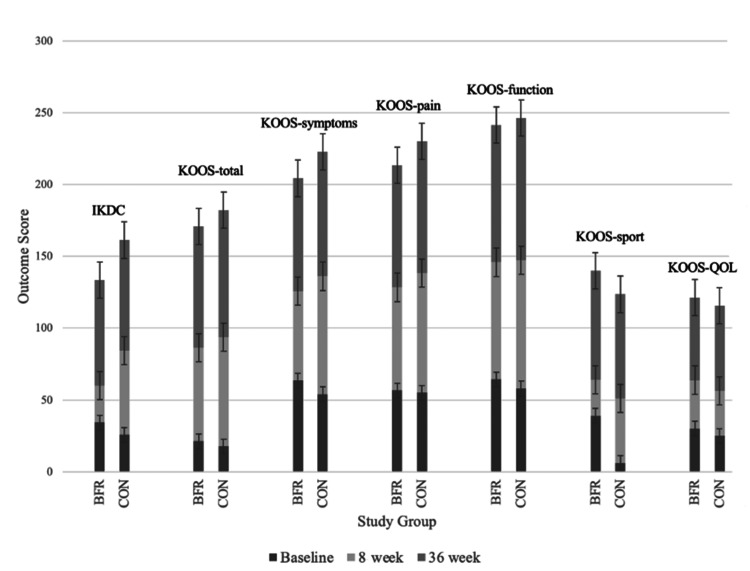
Patient-reported outcomes at baseline and eight and 36 weeks postoperatively for the BFR and CON groups as reported by validated patient-reported outcome tools including IKDC and KOOS BFR: blood flow restriction; CON: control; IKDC: International Knee Documentation Committee; KOOS: Knee Injury and Osteoarthritis Outcome Scores; QOL: quality of life

PROMs were acquired at baseline as well as at eight and 36 weeks postoperatively (Figure 4). At baseline, there were no statistical differences between the BFR and CON groups in terms of PROMs, with total IKDC scores of 34.2±17.5 and 25.7±10.0, respectively (p=0.18), and total KOOS scores of 56.3±21.4 and 47.3±17.7, respectively (p=0.24). Both the IKDC and KOOS scores significantly improved over time for both groups. KOOS-Symptom and KOOS-Sports scores were significantly higher in the CON than the BFR group at eight weeks postoperatively. 

Of the patients in the early BFR group, one patient (patellar tendon autograft) had reports of knee stiffness and lateral knee pain at six months postoperatively, with a knee range of motion of 5-135°. Of the patients in the CON group, one patient had persistent patellofemoral pain and knee swelling at 36 weeks postoperatively. There were no instances of sensorimotor perturbations such as prolonged paresthesia, any cases of arthrofibrosis, or need for revision surgery within either the CON or early BFR cohorts.

## Discussion

The purpose of this pilot study was to compare changes in postoperative quadriceps strength between conventional ACLR rehabilitation with early BFR training and conventional ACLR rehabilitation with sham (e.g., non-therapeutic pressure) BFR training. A secondary purpose was to determine the effect of adding early BFR training on late-stage (36-week) functional outcomes using standard performance tests and PROMs. We found no difference between quadriceps and hamstring strength at eight and 36 weeks postoperatively. MRI muscle volume, MRI adipose composition, single-leg hop scores, and PROMs were similar between groups at the tested time intervals. Both groups improved in strength and functional outcomes over time. An important limitation of this pilot study is that enrollment was limited and our sample size was underpowered for our primary outcome.

Quadriceps volume, strength, and composition

The findings of this study are supported by the current literature. A study by Curran et al. randomized ACLR patients to concentric and eccentric rehabilitation protocols with and without BFR utilization, setting the AOP at 80% and evaluating various outcome measures at set time points: preoperatively, after BFR, and at the return to activities [21]. They found no difference in quadriceps muscle activation, quadriceps peak torque, and rectus femoris muscle volume between any of the four groups at any time points. Hughes et al. randomized subjects to BFR and conventional high-load rehabilitation protocols and found no difference in improvement in quadriceps strength and volume of the operative extremity in their study [22]. These results are in contrast to a study by Ohta et al., which looked at knee flexor and extensor strength in subjects randomized to BFR and conventional rehabilitation protocols and found that at the 16-week postoperative mark, subjects who had undergone the BFR protocol demonstrated increased quadriceps and hamstring strength as well as greater quadriceps muscle group volume compared to subjects who rehabilitated by the standard of care without BFR [23]. The difference in their results could be explained by the relatively longer BFR utilization period (16 weeks versus eight weeks). Along these lines, Li et al. prospectively compared subjects post-ACLR randomized to conventional therapy versus BFR at 40% AOP and BFR at 80% AOP and found that BFR utilization resulted in increased quadriceps volume and strength, with larger improvements noticed in the higher AOP group [24]. Another prospective study by Vieira de Melo et al. found increased quadriceps and hamstring strength after a BFR rehabilitation protocol utilizing 80% AOP, evaluating patients after eight and 12 weeks of rehabilitation [25]. Erickson et al. randomized patients to BFR and standard-of-care post-ACLR rehabilitation protocols and evaluated preoperative and postoperative quadriceps muscle strength via a dynamometer and quadriceps muscle morphology and physiology by MRI and muscle biopsies, respectively [26]. They found no significant differences in any outcome variables measured between the two groups. 

The notable heterogeneity in methodology helps to explain the heterogeneity of results and was a primary motivator for methods used in our study. Many of the studies had no standardized BFR protocol and varied in the type of tourniquet device used to achieve an 80% AOP and varied in the length of BFR application [21-24]. Further, few studies used gold-standard methods to evaluate changes in muscle volume [21,22,24]. Despite improvements in methodology, our study did not find that BFR training positively impacted thigh muscle strength or muscle volume and composition, though our sample size was underpowered, and results could have been confounded by differing graft types and subject BMI between groups. To our knowledge, we are the first to report on adipose composition changes in quadriceps and hamstrings following BFR training post-ACLR. Our study did not demonstrate a significant difference in intramuscular adipose tissue in the quadriceps or hamstring muscle groups between the BFR and standard-of-care study participants. Positive strength changes seen with longer (16 weeks) BFR application suggest that future work to examine whether BFR training incorporated into a standardized rehabilitation protocol with a personalized pressure cuff for at least 16 weeks results in muscle volume, composition, and strength changes following ACLR.

Functional performance

Single-leg hop tests including six-meter hop time, single hop distance, and triple hop distance have shown to be a reliable and reproducible method that has been widely utilized in assessing lower extremity strength and readiness to return to sport [16,27-29]. There have been no previous randomized controlled studies evaluating the possible effect of BFR on single-leg hop tests. In our study, we did not see an improvement in functional performance in patients who underwent early BFR therapy as part of their postoperative ACLR rehabilitation protocol, as measured by validated single-leg hop tests, at either eight or 36 weeks postoperatively. 

Outcome measures

We did not identify a statistical difference in pain as measured by KOOS-Pain patient-reported outcomes, which contrasts with existing literature. Vieira de Melo et al. demonstrated that BFR afforded improved pain and patient-reported outcomes in their subjects, as measured by Lysholm, IKDC, and KOOS scores [25]. Similarly, Li et al. noted improvement in pain scores in BFR subjects [24]. The protocol utilized by Vieira de Melo et al. was 12 weeks, longer than the BFR protocol utilized in our study, which could be a contributing factor to the significant improvements they found in their study, whereas Li et al. utilized a shorter eight-week duration of BFR therapy with adequately powered group sizes and saw improvements in pain scores. Khalil et al. evaluated knee pain by the visual analog scale at six and 12 weeks postoperatively, comparing pain scores between subjects who were randomized to BFR and conventional therapy groups, and found that knee pain improved within both groups with no significant differences between the groups [30]. Recently published meta-analyses of randomized controlled trials comparing BFR therapy to standard of care failed to demonstrate significant differences in patient-reported outcomes with BFR utilization [31,32]. The lack of consistent improvement in pain across BFR studies indicates that there may be an underlying factor unrelated to strength or function that explains why some individuals have reduced pain levels following BFR training. Future studies should more thoroughly examine the effect BFR training may have on pain centers or perception.

Our findings suggest that there is no clear benefit to the addition of short-term (eight-week/200-minute) BFR into standard-of-care ACLR early rehabilitation for knee strength, prevention of muscle atrophy or fatty decomposition, functional outcomes, or patient-reported outcomes. Given the high heterogeneity of BFR protocols reported in the literature and our low statistical power, our results should only be interpreted with caution for ACLR rehabilitation. Future studies should build upon our work by using our standardized approach but with larger sample sizes and extended periods of BFR training. We hope that by adding specific details such as those in our protocol, we can help standardize the use of BFR postoperatively. At present, we recommend that the theoretical benefits of BFR should be viewed with caution and that surgeons should counsel patients that there are inconsistent reported results regarding BFR rehabilitation in comparison to standard-of-care protocols.

There are several limitations of our study that warrant consideration. First, due to a variety of factors, there was an underpowered final sample size of five patients per group rather than 12. This study was conducted during the COVID-19 pandemic, which could have played a role in limiting follow-up. Future investigators may consider a simpler postoperative regimen or more expansive inclusion criteria to improve patient enrollment and decrease exclusion from insurance restrictions or other logistics of therapy. Second, there was no one standard graft type utilized for ACLR in our study subjects. There was not an even distribution of hamstring and extensor mechanism autografts between groups. It may be that one type of graft responds better to BFR training than others; however, our limited sample size prevented us from exploring this concept. Third, we used 200 minutes to standardize BFR training rather than a time period (e.g., eight weeks). As a result, the time to complete 200 minutes varied across participants. It may be that consistent dosage rather than absolute dosage is critical to eliciting positive changes with BFR training. Our small size limited us from exploring time to complete BFR training as a statistical variable. Future studies should examine both total BFR minutes and time to complete BFR training. Another limitation in our patient demographics is that the mean BMI differed between the BFR and CON groups, which served as a confounder despite randomization and could have affected muscle strength, adiposity versus lean mass, and functional outcomes. Furthermore, physical therapists were not blinded to the study group of each participant, as it was necessary for them to be able to set cuffs at the appropriate AOP; hence, this represents another limitation in our study. Lastly, we did not calculate an intra-observer reliability value for our single examiner that calculated MRI measurements; we list here as a limitation, but previous literature has shown that the MRI measurement method utilized in our study has high intra-observer reliability [19].

## Conclusions

In our pilot study, we did not find significant improvements in quadriceps and hamstring strength between the BFR and CON groups by eight weeks postoperatively. We also did not find a difference in MRI-assessed quadriceps and hamstring muscle volume and intramuscular adipose over time or between the BFR and standard-of-care groups. Thus, we did not observe a clinical benefit of short-term BFR training done early in ACLR rehabilitation on thigh muscle strength, volume, or composition. As this pilot study was underpowered and subjects were not randomized by graft selection, larger studies with graft randomization but otherwise similar methodology are warranted to better understand the potential effects of early BFR on patient rehabilitation after ACLR.
